# State-Granted Practice Authority: Do Nurse Practitioners Vote with Their Feet?

**DOI:** 10.1155/2012/482178

**Published:** 2012-11-19

**Authors:** John J. Perry

**Affiliations:** Economics Program, Centre College, 600 West Walnut Street, Danville, KY 40422, USA

## Abstract

Nurse practitioners have become an increasingly important part of the US medical workforce as they have gained greater practice authority through state-level regulatory changes. This study investigates one labor market impact of this large change in nurse practitioner regulation. Using data from the National Sample Survey of Registered Nurses and a dataset of state-level nurse practitioner prescribing authority, a multivariate estimation is performed analysing the impact of greater practice authority on the probability of a nurse practitioner moving from a state. The empirical results indicate that nurse practitioners in states that grant expanded practice are less likely to move from the state than nurse practitioners in states that have not granted expanded practice authority. The estimated effect is robust and is statistically and economically meaningful. This finding is in concert with and strengthens the wider literature which finds states that grant expanded practice authority to nurse practitioners tend to have larger nurse practitioner populations.

## 1. Introduction

Nurse practitioners (NPs) are, according to the International Council of Nurses, “a registered nurse who has acquired the expert knowledge base, complex decision-making skills, and clinical competencies for expanded practice, the characteristics of which are shaped by the context and/or country in which s/he is credentialed to practice” [1]. In the United States, NPs are typically masters-prepared registered nurses and have become an increasingly important part of the health care system. They have over time obtained greater practice authority through state-level regulatory changes which has fundamentally altered what an NP can do as a caregiver. This has, in turn, altered their role in the health care system. In particular, these changes have allowed NPs to take a more central, independent role in providing health care. While NPs were initially seen as “physician extenders” by the wider health care industry in the United States, they have become, in many respects, “physician replacers.” Today, in most U.S. states, NPs can see, diagnose, prescribe, and in general provide care for patients as a general practice physician would. As such, these regulatory changes in practice authority, and the “rise” of the NP they have ushered in, have fundamentally changed the NP labor market. 

As would be expected in an industry as important as health care, the “rise of the NP” has been accompanied by a large body of research. In general, this research can be grouped into four broad categories: their rise as caregivers, the cost and quality of NP care, NP populations, and NP labor market outcomes.

The first body of research details the NP's rise as a primary caregiver. This body, of work traces the origins of the NP in the U.S., their history, and the current role of NPs in the health U.S. care industry [2–6].

The second and the largest and most active line in the literature investigates the quality and cost of care NPs provide. A primary finding that can be drawn from this literature is that care provided by NPs is nearly outcome-indistinguishable to that of physicians [7–12]. In addition, the research shows that care provided by NPs tends to receive at least as high patient satisfaction ratings as that of physicians [13–15]. This branch of the literature also gives compelling evidence that beyond providing quality care, NP care is also cost effective [16, 17].

A third branch of the literature, and one that is directly pertinent to this research, examines how regulatory changes to NP practice authority have impacted total NP populations in states. Sekscenski et al. [18] found, using an index measure of state-level practice authority granted to NPs, states which granted greater practice authority tended to have larger populations of NPs than those that did not. The United States Department of Health and Human Services [6], expanding on Sekscenski et al., also found that the level of practice authority granted was correlated with increased NP populations. Kalist and Spurr [19], using a regression framework, found that states that had granted NPs greater practice authority had larger enrolments in masters nursing programs, all else equal. 

The fourth and smallest branch of the literature on NPs examines the impact of regulatory changes in NP practice authority on their own labor market outcomes. Dueker et al. [20] made an early contribution to this literature and found the unintuitive result that greater practice authority leads NPs to have lower incomes. Perry [21], using a richer data set in which NPs can be specifically identified, a shortcoming of Dueker et al. work, finds that NPs who are granted greater practice authority experience significant increases in their incomes relative to NPs who are not granted greater practice authority. 

The current project sits at the nexus of the third and fourth bodies of literature. No research that the author is aware has taken a broad, microlevel approach and examined individual NP location responses to state-level regulation. This research does just that by examining the impact of state-granted practice authority on individual NP migration choices. 

Using a national sample of NPs spanning 1991 to 2003, a period of significant state-level change in NP regulation, this paper finds that NPs do “vote with their feet.” In specific, an NP in a state that has granted greater practice authority to NPs is less likely to move from the state than otherwise. This result is in concert with—and helps explain—the larger macrolevel literature that practice authority expansions are associated with greater NP populations in a state as well as the research on the economic impact to NPs of authority expansions.

## 2. Methods 

There are many reasons an NP could choose to move from one state to another. Since the work an NP is allowed to perform is governed by the authority a state grants, it is reasonable to expect the level of practice granted by a state would impact any move decision, even if at the margin. If practice authority is important, one would expect to see NPs “vote with their feet,” all else equal. If practice authority is not substantially important, NP moves would not be responsive and move rates would be largely unaffected by changes in state-level practice authority. In either case, the question is an empirical one and policy is directly informed.

A straightforward empirical model that estimates the impact of expanded practice authority on a NP's likelihood to move while controlling for other confounding factors is as follows:

(1) P(movei,s,t=1 ∣ controls)=α+NPAuthoritys,t∗β+Xi,s,t∗∂+ηs+θt+ui,s,t,

where move is a dichotomous variable that equals “1” if the *i*th NP moves from state *s* in year *t* and “0” otherwise. NPAuthority is a measure of NP practice authority in a state, equal to “1” if the authority is present in state *s* in year *t* and “0” otherwise. *X* is a matrix of personal characteristics of the *i*th NP in state *s*in year *t*. *θ*_*t*_ is a vector of year dummies to control for year-specific differences and *η*_*s*_ is a vector of state fixed effects. 

Equation (1) is estimated both as an Ordinary Least Squares linear probability model and a probit model where the dependent variable is set to zero if the NP did not move and one if the NP moved from one state to another. 

The data used in the analysis comes from two sources. The first is the National Sample Survey of Registered Nurses (NSSRN). The NSSRN is a probability sample of the universe of Registered Nurses (RN) in the United States and is conducted every four years by the U.S. Department of Health and Human Services. While the focus of the survey is the RN population, NPs are included and identifiable in the data. The NSSRN observation level is the individual and contains a variety of demographic, geographic, and professional variables. The NSSRN sample years included in this research are 2004, 2000, 1996, and 1992 which corresponds nicely to a large wave of change in state-granted NP practice authority.

Critical to this study, the NSSRN has information on the state the NP lived in during the year of the survey as well as where the NP lived the* previous* year. While the combined NSSRN data is a repeated cross-section, the questions about where the NP lived in the year of the survey and where the NP lived the year prior provides the opportunity to “see” where an individual lived in two contiguous years. This yields a unique opportunity to “see” an individual NP move or, just as important, not move. A total of 4,103 NPs are included in the sample aged from 26 to 64.  Table 1 provides summary statistics for the sample.

With data on location and demographics of individual NPs, some measure of state-level practice authority is needed. This study follows the larger literature on NPs and uses the level of prescriptive authority granted as a general measure of NP practice authority a state grants. Specifically, whether or not a state grants NPs some level of controlled substance prescriptive authority is used.

While controlled substance prescriptive authority is an admittedly imperfect measure of NP authority, it is a widely used component of practice authority in the literature [18, 19, 21]. It also, in a single measure, provides an intuitive and tractable measure of the authority an NP enjoys in a state. 

A by year, by state database of state regulation on controlled substance authority for NPs was compiled by the author through a review of the annual “Legislative Update” of the journal *Nurse Practitioner* by Pearson [22–25] and supplemented with research of individual state statues. This data was used to create a dichotomous variable that was equal to one if the state allowed NPs some level of controlled substance prescriptive authority and zero if it did not for each year. 

Table 2 provides a snapshot of the number and percent of states authorizing NPs to prescribe controlled substances by year, from 1991 to 2003. As can be seen from Table 2, there was a significant change in the proportion of states that authorized NPs to have controlled substance authority. This variation in state practice authority makes the time period ideal to investigate.

## 3. Results 

The results of the regression estimations can be found in Table 3. For the probit estimation, the marginal effects are reported since probit coefficient estimates are not directly interpretable. The interpretation of the marginal effect coefficient is the change in the probability of a move for an NP with the sample mean characteristics if there is a one unit change the independent variable in question. 

All of the demographic variable coefficients are in line with expectations and most are statistically significant. Of most interest is that the estimated impact of state practice authority is negative and significantly different than zero at conventional significance levels. This is true for both the linear probability model and the probit model which provides some robustness check. 

The interpretation is that an NP is less likely to move from a state that has granted expanded prescriptive authority than if the state had not, controlling for other influences. Not only is the effect statistically significant, it is also material. The point estimate from both estimates is approximately −0.03. This implies that if a state has granted NPs expanded prescriptive authority, the probability of an average NP moving from the state falls by roughly three percentage points. Considering that on average about 6.5% of NPs in the sample moved in a given year, a state authorizing expanded authority to NPs leads to a reduction in the probability of moving of around 46%. This implies that the level of authority a state grants to NPs is meaningful to NP locational decisions.

It is also informative that the estimation results are robust to changes in specification and sample. The estimated results are materially unchanged when age restrictions and/or demographic variables are changed or omitted. The robustness of the empirical estimates provides some assurance that the effect of NP expanded prescriptive authority being measured is real.

There are weaknesses in the current research that should be acknowledged. Of particular note, while the NSSRN has high level of detail on an individual NP, the information is for the specific survey year. For example, the 2004 NSSRN data asks the respondent about the status in 2004 of most variables, such as income and marital status. Since the NSSRN asks where the NP was in the previous year, the the data allows us to “see” what state the NP lived in 2003 which in turn allows us to see an NP move. Unfortunately, we do not “see” many other variables of note in 2003. This limits the controls that can be included in the regression. Most of the independent control variables that were included are variables that can be known or inferred from year to year (sex, age, race). Marital status and whether the NP has a child at home were also included in the final specifications even though they are reported only in the current year and not the previous year. That the coefficient estimates are as expected and the model is robust to whether these demographic variables are included are not provides some reassurance that the measured impact of NP authority is valid and not adversely impacted.

There is also the limitation as to the measure of state practice authority. Whether or not a state allows NPs to prescribe controlled substances was the measure employed but there are a number of reasonable approaches to measuring a state's practice environment. However, there is no definitive measure. The current measure is commonly used in the literature as well as intuitive, tractable and represents a clear measure of difference between states as to what NPs are authorized to do as caregivers. It is not, however, a perfect measure of NP authority.

## 4. Conclusion

This research provides the first broad, microlevel analysis of the impact of state-regulated practice authority changes on individual NPs' migration choices. The core finding is that an NP in a state that has granted expanded practice authority as measured through controlled substance prescriptive authority is less likely to move than if the state had not granted such authority. This finding is robust to specification and estimation technique. 

This finding is in line with the macrolevel literature that finds a positive correlation between expanded practice authority and NP populations. In fact, it strengthens the macrolevel literature by providing a likely mechanism for which populations of NPs can change between states in response to state-level regulatory changes. Coupled with the research literature on quality and cost of care, which generally finds NPs provide care clinically similar to same-level physician-provided care, the results are informative to policy makers interested in the effects of regulatory changes on NP practice authority on the health care industry. This research also suggests that for regulated occupations, which include nearly all medical occupations, regulation changes of practice authority can materially impact individual behaviour.

## Figures and Tables

**Table 1:** Sample summary statistics.

Variable	Mean	Std. dev.	Min	Max
Move	0.065	0.247	0	1
NPs have controlled substance prescriptive authority	0.684	0.465	0	1
Married	0.734	0.442	0	1
Male	0.051	0.220	0	1
White	0.891	0.312	0	1
Child at home	0.394	0.489	0	1
Age	44.549	8.624	26	64

*n* = 4103.

Years: 1992, 1996, 2000, and 2004.

**Table 2:** Number and percent of states granting NPs controlled substance prescriptive authority.

Year	States	Percent
2003	45	88%
1999	37	73%
1995	29	57%
1991	15	30%

**Table 3:** NP's move regression selected results.

	OLS		Probit	
	Coef.	Std. Err.	Coef.^~^	Std. Err.
NPs have controlled substance prescriptive authority	−0.030*	0.014	−0.032*	0.017
Married	−0.026*	0.010	0.024*	0.009
Male	0.060*	0.023	0.060*	0.023
White	0.003	0.013	0.002	0.011
Age	−0.015*	0.005	−0.011*	0.003
Age squared	0.000*	0.000	0.000*	0.000
Child at home	−0.028*	0.010	−0.024*	0.008

*Significant at 5% level.

^~^Probit coefficient estimates are reported as marginal effects for comparison purposes.

Note: NPs between 26 and 64 are included. Year and state fixed effects are incorporated. Standard errors are robust.
